# Lessons from COVID-19-free Vanuatu: intensive health operations for Phase 1 of repatriation and quarantine, May–July 2020

**DOI:** 10.5365/wpsar.2020.11.4.004

**Published:** 2021-03-10

**Authors:** Posikai Samuel Tapo, Tessa B Knox, Caroline van Gemert-Doyle, Obed Manwo, Edna Iavro, Wendy Williams, Rosaria Maurice, Griffith Harrison, Matthew Cornish, Michael Benjamin, Vincent Atua, Jimmy Obed, Geoff Clark, Philippe Guyant, Basil Leodoro, Len Tarivonda

**Affiliations:** a Vanuatu Ministry of Health, Port-Vila, Vanuatu.; b Vanuatu Country Liaison Office, World Health Organization, Port-Vila, Vanuatu.; c Vanuatu Health Program, Port-Vila, Vanuatu.; d School of Population and Global Health, The University of Melbourne, Melbourne, Australia.; e Burnet Institute, Melbourne, Australia.

## Abstract

International borders to Vanuatu closed on 23 March 2020 due to the global COVID-19 pandemic. In May–July 2020, the Government of Vanuatu focused on the safe and timely return of citizens and residents while ensuring Vanuatu remained COVID-19 free. Under Phase 1 of repatriation, between 27 May and 23 June 2020, 1522 people arrived in the capital, Port Vila, and were placed in compulsory government-mandated 14-day quarantine in 15 hotels. Pre-arrival health operations included collection of repatriate information, quarantine facility assessments, training for personnel supporting the process, and tabletop and functional exercises with live scenario simulations. During quarantine, health monitoring, mental health assessments and psychosocial support were provided. All repatriates completed 14 days of quarantine. One person developed symptoms consistent with COVID-19 during quarantine but tested negative. Overall health operations were considered a success despite logistical and resource challenges.

Lessons learnt were documented during a health sector after-action review held on 22 July 2020. Key recommendations for improvement were to obtain timely receipt of repatriate information before travel, limit the number of repatriates received and avoid the mixing of “travel cohorts,” ensure sufficient human resources are available to support operations while maintaining other essential services, establish a command and control structure for health operations, develop training packages and deliver them to all personnel supporting operations, and coordinate better with other sectors to ensure health aspects are considered. These recommendations were applied to further improve health operations for subsequent repatriation and quarantine, with Phase 2 commencing on 1 August 2020.

## Problem

**Figure 1:**
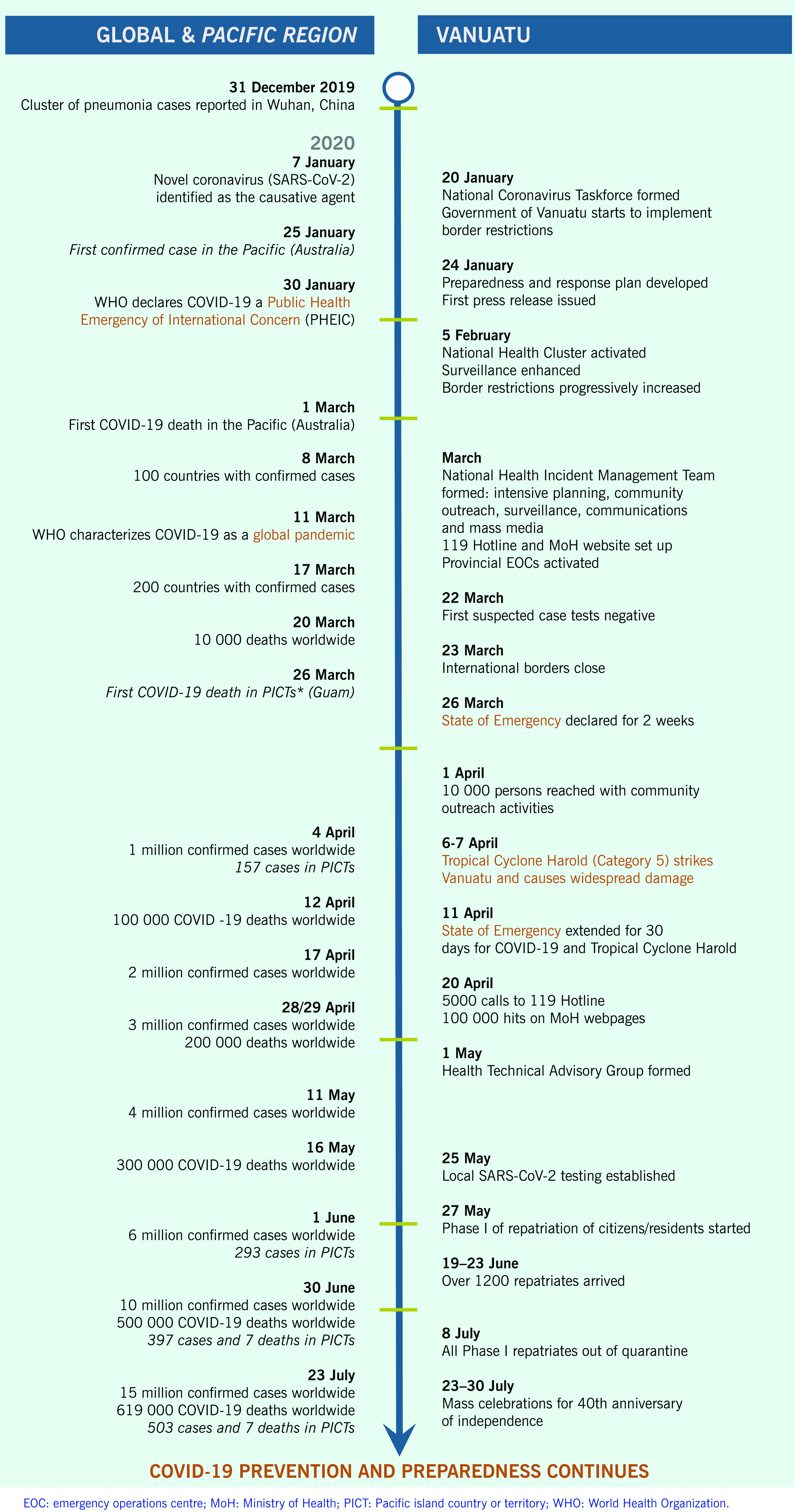
Timeline of the COVID-19 pandemic globally, in the Pacific and in Vanuatu and beyond, 31 December 2019 to 30 July 2020

Vanuatu, in the South Pacific, comprises 83 islands with a total population of around 307 000. As of 1 August 2020, it was one of the few countries with no confirmed cases of coronavirus disease 2019 (COVID-19), (*1*) mainly due to international border restriction measures implemented from late January 2020 and full border closure from 23 March 2020. A State of Emergency (SoE) was declared on 26 March 2020 (*2*) to strengthen prevention and containment measures in response to the COVID-19 global pandemic (Fig. 1). The SoE was extended to include Tropical Cyclone Harold, which struck Vanuatu on 6–7 April 2020, (*3*) and was subsequently extended to 31 December 2020. (*4*)

[**insert** Figure 1]

Many Vanuatu citizens and residents travel or reside overseas, particularly under seasonal work programmes in Australia and New Zealand, or for study in Fiji and New Caledonia. As the global COVID-19 pandemic affected work and study abroad, many of these expressed interest in being repatriated to Vanuatu.

## Context

The Government of Vanuatu undertook bilateral negotiations to ensure the safe and timely return of priority citizens and residents. Phase 1 of the operations (May–July 2020) aimed to ensure that priority repatriation and quarantine were completed ahead of the celebrations for the 40th anniversary of independence (23–31 July 2020). Phase 2 of the operations started after these celebrations.

## Actions

Between 27 May and 23 June 2020, 1522 returning citizens and residents arrived through the Phase 1 repatriation operation. Fourteen flights were received in the capital, Port Vila, from Solomon Islands (1), Fiji (1), the Philippines (1), New Zealand (8), New Caledonia (1) and Australia (2), with 11 of these flights arriving between 19 and 23 June 2020.

In accordance with the Vanuatu Public Health Act [Cap. 234], (*5*) mandatory quarantine in government-designated hotel facilities was instituted for all arriving repatriates. Quarantine was for a 14-day period, based on technical recommendations from the Vanuatu Health Technical Advisory Group (*6*) and the incubation period of severe acute respiratory syndrome coronavirus 2 (SARS-CoV-2). The health operation activities are summarized in Table 1 and Fig. 2.

**Table 1:** Purpose and description of health operation activities for Phase 1 of repatriation and quarantine in Vanuatu, May–July 2020

Stage/activity	Purpose
**Coordination and staffing**	
**Operations coordination**	To ensure coordination and communication across teams involved in health operations through daily meetings, debriefings and situation reports.
**Pre-arrival preparations**	
**Quarantine facility assessments**	To ensure quarantine facilities selected meet minimum MoH/WHO standards to support compliance while maintaining good health and well-being of repatriates.
**Quarantine facility training and information**	To educate managers and staff on quarantine SOPs, including appropriate use of PPE.
**Other training**	To educate others supporting the repatriation and quarantine process (border security, police, drivers and others) on general COVID-19 information and quarantine SOPs.
**Tabletop and live scenario simulations**	To replicate processes to identify potential issues and areas for improvement, including pre-arrival, transfer from airport to quarantine facilities and registration.
**Procurement and issuance of PPE**	To provide PPE (gloves, masks, gowns, eye protection and hand sanitizer) to those who require it, in accordance with MoH guidance.
**Before departure from origin**	
**Information to repatriates**	To enable appropriate preparation for travel and quarantine.
**Information to MoH**	To inform health operations team preparations based on passenger information (age, pre-existing health issues or medical conditions, and medication requirements).
**Pre-boarding screening**	To collect (through a passenger health declaration form) travel history and ensure repatriates are fit to travel.
**Upon arrival at the airport in Vanuatu**	
**Information to repatriates at border**	To provide further educational information, including through videos screened in the arrival hall.
**Health screening**	To check for signs or symptoms of COVID-19 and review information on passenger health declaration forms before transferring them to quarantine facilities.
**Assigning to quarantine facilities**	To ensure all those who may require specialized medical or health support can access it.
**Luggage and transportation logistics**	To support smooth operations and ensure the comfort of repatriates (including access to required medication).
**Check-in to quarantine facilities**	
**Quarantine order**	To provide medical authorization for placing persons in quarantine through quarantine admission letters.
**Quarantine facility registration**	To collect health and other information to enable admission to quarantine, including through interviews and forms used for subsequent daily health assessments and health clearance.
**Orientation briefing**	To provide repatriates with additional information to support the quarantine process, including advisories from health, hotel and security staff.
**During quarantine**	
**Daily health screening**	To rapidly detect any COVID-19 symptoms (including fevers through measurement of temperature) or other medical issues throughout the quarantine period.
**Other health and medical support**	To provide repatriates with any additional support needed for health and well-being, with a 24/7 nurse and doctor on roster.
**Evaluation of possible COVID-19 cases**	To ensure the detection of COVID-19 cases by testing repatriates who fulfilled the WHO case definition (or others in exceptional circumstances).
**Psychosocial surveys and support**	To detect or assess any mental health or other issues to provide timely support, through surveys including an adapted Kessler Psychological Distress Scale on days 3 and 7 of quarantine.
**Other support**	To provide further support to children or others in quarantine with additional needs (e.g. providing activity packs for children and other services such as currency exchange).
**Incident reports and health risk assessments**	To facilitate rapid reporting of any incidents and inform follow-up or mitigating actions through a standard online incident log system and health risk assessment process.
**Quarantine discharge**	
**Health clearance letters**	To provide medical authorization for discharge from quarantine following 14 days of monitoring, with clearance by a medical officer.
**Pre-discharge debriefing**	To provide final health and other information to repatriates before their departure from quarantine.

MoH: Ministry of Health; PPE: personal protective equipment; SOP: standard operating procedure; WHO: World Health Organization.

Twenty people involved in Phase 1 of the health operations from the Ministry of Health (MoH) and development partner organizations participated in an after-action review (AAR), held on 22 July 2020. The AAR was facilitated by the World Health Organization (WHO) Vanuatu Country Liaison Office, with five observers (WHO staff and senior MoH staff) and three rapporteurs. This report presents the main observations and lessons learnt from Phase 1.

### Main observations and lessons learnt

#### Coordination and staffing

The health operations team comprised staff from the MoH and development partner organizations, with existing staff repositioned or additional personnel contracted to meet critical gaps. A total of 34 provincial public health staff (public health officers, nurses and medical officers) were involved in daily monitoring across the 15 government-designated quarantine facilities, with staff redeployed from other services. The team was highly motivated to support the quarantine process during Phase 1, and continual improvements were made. However, the rapid influx of repatriates over a 5-day period, and the requirement to register and then monitor each person daily with in-person temperature and symptom checks, added a considerable burden to the public health system. The AAR therefore recommended that, for Phase 2, human resource requirements to support operations should be clearly mapped out and options should be identified for surge capacity (e.g. those working in provincial offices, retired staff or new recruits), with opportunities provided for upskilling. The AAR strongly recommended limiting the number of arriving repatriates and those in quarantine to a manageable number, based on staff numbers and availability of quarantine facilities.

During Phase 1, separate daily meetings were held at both the MoH and Shefa Community Health Services, during which staff provided updates on operations, identified or were notified about current health issues, and determined the actions required. The AAR highlighted the need to strengthen coordination of health operations between the MoH and provincial health offices, to avoid replication and undue burden on managers, and to ensure joint daily briefings, debriefings and production of consolidated situation reports. Tabletop and functional exercises with live scenario simulation exercises were conducted with national and provincial staff throughout Phase 1, in parallel with ongoing repatriation operations. They covered arrival, transfer from airport to quarantine facilitiess and registration – lessons learnt were fed back for continuous improvement.

#### Pre-arrival preparations

In Phase 1, the MoH requested repatriate information (e.g. age, sex, health issues, medical conditions and required medication) from foreign missions in advance of travel, but little information was provided. This constrained preparatory work by the health operations team (e.g. pre-arrival quarantine facility allocations). The AAR recommended that the MoH develop an electronic system to collect repatriate information 72 hours before travel to enable MoH assessment of epidemiological risk and health approval before travel; preparation for quarantine, including pre-allocation to quarantine facilities based on health and medical needs; and systematic registration and tracking of all arriving repatriates.

The AAR also highlighted the need to strengthen coordination with the other agencies involved in repatriation planning and execution, such as the Department of Foreign Affairs and the National Disaster Management Office (NDMO).

In Phase 1, the MoH developed selection criteria to guide the identification of suitable quarantine facilities. Criteria included status of services (running water, hot water, electricity, phone, Internet and television) and spacing between beds, potential to open windows for airflow, pathways around the quarantine facility, and outdoor space available for movement and exercise, accessibility to emergency or other medical care, and logistics for daily monitoring. However, the selection of facilities was led by the NDMO rather than the MoH. The AAR recommended that the MoH define clear selection criteria for quarantine facilities and decide which facilities would be appropriate for Phase 2.

To minimize costs, one to six people were allocated to a room in Phase 1, with people from different travel origins sometimes housed together, which the MoH identified as an elevated transmission risk. The AAR therefore recommended that, for Phase 2, no more than two people should share a room, and “travel cohorts” should be maintained by allocating people from a particular travel origin and plane to a single quarantine facility (unless access to specialized medical care was needed).

The issues identified with quarantine facility selection and room allocation highlighted the importance of multisectoral coordination, and the need for the MoH to actively engage with the NDMO, to enable appropriate planning and operations that consider health risk factors, logistics and cost.

#### Before departure from origin

Information on the process, requirements and restrictions for quarantine was provided to repatriates in a pre-travel information note issued by the Director of Public Health, enabling repatriates to adequately prepare mentally and logistically for quarantine. However, surveys conducted during quarantine indicated that not all repatriates received adequate information before departure, and people were frustrated, mainly due to unclear or conflicting information on access to tobacco and alcohol products, and kava. The information was provided primarily through Vanuatu overseas missions, which may not have received accurate information and which were not available in some countries from which people travelled. The AAR therefore recommended that consistent pre-travel information be issued to repatriates well in advance of travel, to clearly communicate quarantine rationale, processes and restrictions, and consequences of non-compliance.

#### Upon arrival at the airport in Vanuatu

Quarantine admission letters were issued by the Director of Public Health, in line with the Public Health Act. (*5*) In Phase 1, arriving repatriates completed a paper passenger health declaration form, providing information on travel or contact history, signs and symptoms of COVID-19 or any other health issues or conditions. Completed forms were evaluated by Shefa Community Health Services to determine whether repatriates required specialized quarantine conditions, although the criteria for such exceptions were unclear. Feedback from the health operations team highlighted the lack of a clear process if a repatriate with no symptoms was found to have a temperature over a pre-established threshold. The AAR therefore recommended updating standard operating procedures for arrival health screening.

Upon arrival or registration, issues were identified for 42 people; these included medication requirements (41), pregnancy (14), allergies (30), addiction (*5*) and disability. (*3*) The AAR recommended that allocation to quarantine facilities consider pre-existing health conditions or issues, travel origin and travel history.

#### Check-in to quarantine facilities

Data from passenger health declaration forms were later entered into a database; this process led to delays in data availability and constrained use of the data for quarantine operations. Separate registration forms were required for check-in to quarantine facilities and were again reviewed by Shefa Community Health Services. The AAR recommended improving information collection and management by using tablets for onsite data entry, updating online forms, and developing dashboards for rapid and clear communication and action.

#### During quarantine

Daily health screenings included assessment of self-reported symptoms and measurement of temporal temperature by infrared thermometer to detect fever. In Phase 1, only one person was identified with symptoms consistent with the WHO case definition for COVID-19 at the time. (*7*) A nasopharyngeal swab was found to be negative for SARS-CoV-2; the person was discharged following 14 days of quarantine and was later reclassified as not having been a suspected case. Health screenings also identified other health issues (e.g. foodborne illness and dental issues). All those in quarantine were cleared for discharge on day 15 after their arrival (to ensure a full 14 days in quarantine).

A total of 2480 quarantine-experience surveys and 2098 Kessler Psychological Distress Scale assessments were conducted around days 3 and 7 of quarantine. The aim was to conduct two surveys for each individual, but limited numbers of trained health staff meant this was not always possible. Pooled results indicated that quarantine was “easy” for 78%, “a bit difficult” for 20% and “very difficult” for 2% of respondents. Overall, eight individuals showed signs of moderate or severe distress. At some facilities, there was dissatisfaction with the amount of time allocated for exercise or physical activity, or with special dietary requirements (e.g. food allergies or religion) not being adequately met. Almost all (99%) repatriates felt safe during quarantine and 92% knew who to contact for any health issues, but 16% were worried about their safety after discharge from quarantine. Consultations were held with community leaders in areas to which repatriates were to return, to address concerns and promote understanding that those discharged from quarantine did not pose a health risk to the population. The AAR recommended proactive community engagement to reduce stigma towards those discharged from quarantine, and follow-up psychosocial monitoring for those discharged from quarantine.

A total of 43 incidents were logged using the MoH system. Most were related to non-health incidents (e.g. losses or delays with luggage); others related to the absence or behaviour of quarantine support staff, including non-compliance with quarantine restrictions. Two instances of breaches of procedures and protocols by quarantine staff triggered health risk assessments. Both incidents led to hotel staff being quarantined, even though this was not in line with MoH recommendations. The AAR noted that a streamlined quarantine facility incident reporting and health risk assessment system is imperative to address issues rapidly and to mitigate risks. The AAR also recommended that the Public Health Act be revised to adequately reflect directed quarantine, and to enable penalties for breaches of quarantine directives, either by those in quarantine or by other members of the public.

Personal protective equipment (PPE), including gloves, masks, gowns, eye protection and alcohol sanitizer, were issued in accordance with MoH guidelines to all staff working in quarantine facilities and airports and to drivers and boat operators involved in transporting repatriates to quaratine facilities. There were some reports of inappropriate use of this PPE (e.g. unnecessary use of gloves). Although 695 people attended some 29 formal training sessions, not everyone supporting repatriation and quarantine processes received training; this led to differences in how protocols were applied and standards were maintained (e.g. PPE use and the amount of time allowed for daily exercise). The AAR therefore recommended that a full and comprehensive training package be developed to include content tailored for various staff supporting the quarantine process.

Other support during quarantine included provision of activity and entertainment packs to children and services such as exchange of currency and shopping. The health operations team also liaised with hotel management on health-related issues; the AAR recommended that this additional role be clearly defined for Phase 2.

#### Quarantine discharge

Some delays were experienced as repatriates went through health clearance and discharge from quarantine, due mainly to the limited availability of medical officers to appraise health data. The AAR therefore recommended improved quarantine discharge and additional registered medical officers to assess and sign discharge summaries. Before discharge, repatriates were debriefed on actions to take if COVID-19 signs and symptoms were observed, and to thank them for their cooperation that enabled a successful quarantine process.

## Conclusion

Health operations instituted in Vanuatu to support government-managed repatriation and quarantine from May to July 2020 were successful. Challenges included a lack of information to guide health planning, high volumes of arrivals, insufficient health staff, poor consideration of health factors for quarantine facility selection and allocations, and inadequate multisectoral coordination. Lessons learnt from health operations in Phase 1 were documented during an AAR in late July 2020. The recommendations will be applied to Phase 2 (from August 2020).

## Ethics statement

The Vanuatu Health Ethics and Research Committee advised that ethics approval was not required because data were collected as part of the pandemic response and in line with the Vanuatu Public Health Act of 1994, with only non-identifiable data collated.
